# Physicochemical Properties and In Vitro Antioxidant Activity Characterization of Protein Hydrolysates Obtained from Pumpkin Seeds Using Conventional and Ultrasound-Assisted Enzymatic Hydrolysis

**DOI:** 10.3390/foods14050782

**Published:** 2025-02-25

**Authors:** Ana Flávia Coelho Pacheco, Flaviana Coelho Pacheco, Jeferson Silva Cunha, Gabriela Aparecida Nalon, Jhonathan Valente Ferreira Gusmão, Fábio Ribeiro dos Santos, Irene Andressa, Paulo Henrique Costa Paiva, Alline Artigiani Lima Tribst, Bruno Ricardo de Castro Leite Junior

**Affiliations:** 1 Instituto de Laticínios Cândido Tostes, Empresa Agropecuária de Minas Gerais (EPAMIG), Tenente Luiz de Freitas, 116, Juiz de Fora 36045-560, MG, Brazil; ana.pacheco@epamig.br (A.F.C.P.); paulohcp@epamig.br (P.H.C.P.); 2 Department of Food Technology (DTA), Federal University of Viçosa (UFV), Viçosa 36570-900, MG, Brazil; flaviana.pacheco@ufv.br (F.C.P.); jeferson.cunha@ufv.br (J.S.C.); gabriela.nalon@ufv.br (G.A.N.); jhonathan.gusmao@ufv.br (J.V.F.G.); fabio.r.santos@ufv.br (F.R.d.S.); irene.andressa@ufv.br (I.A.); 3 Núcleo de Estudos e Pesquisas em Alimentação (NEPA), Universidade Estadual de Campinas (UNICAMP), Campinas 13083-852, SP, Brazil; tribst@unicamp.br

**Keywords:** emerging technologies, commercial proteases, proteolysis, food applications, functional characteristics, peptides

## Abstract

Pumpkin seed proteins (PSPs) are a promising resource for obtaining bioactive peptides but their low solubility hinders enzymatic hydrolysis, reducing yield and bioactivity. In addition, enzymatic processes require specific conditions and long processing times; improving the efficiency of this process is essential to expand its industrial applications. In this context, using a high-frequency, low-intensity ultrasound (US) has proven to be an effective strategy for optimizing the hydrolysis of plant protein. This study evaluated the US-assisted (38 W/L, 40 kHz) and conventional hydrolysis of pumpkin seed proteins (PSPs) for 180 min at 25 °C, 40 °C, and at the optimum temperature condition for each enzyme studied (60 °C for Brauzyn^®^, 55 °C for Flavourzyme^®^, and 50 °C for Neutrase^®^), as well as the impact of this process on the macrostructural and functional characteristics of the hydrolysates obtained. The degree of hydrolysis (DH) was significantly higher in US-assisted reactions, reaching increases of up to 57.7% with Neutrase^®^ at 40 °C. The US also positively influenced the protein solubility of the hydrolysates, especially at pH levels close to the isoelectric point, with improvements of up to 100%, compared to the hydrolysates obtained from the conventional reaction. The antioxidant activity was also enhanced by the US, compared to the conventional reaction, emphasizing the hydrolysates obtained through the action of Flavourzyme^®^, which showed increases of 52.4% and 42.6% in the scavenging of DPPH and ABTS radicals, respectively. The analysis of the mean particle size revealed significant reductions with the US (<26.2%). Consequently, the polydispersity index (PDI) demonstrated greater uniformity in the particles obtained from the US-assisted reactions (reductions of up to 20.3%). UV-Vis spectroscopy and intrinsic fluorescence also indicated possible alterations in the tertiary structure of the peptides obtained, mainly in US-assisted reactions. Therefore, US-assisted PSP hydrolysis resulted in better enzymatic performance and produced protein hydrolysates with bioactive potential for food applications.

## 1. Introduction

In the recent years, plant-based proteins have gained prominence for being more environmentally friendly, sustainable, and healthy [1]. The production and consumption of plant-based proteins align with the United Nations’ Sustainable Development Goals for 2030 and reflect the current market trends focused on cleaner labels [2,3]. Plant-based proteins can also be used to produce peptides with intrinsic biological properties, such as antioxidant activity [4,5,6,7].

Antioxidant peptides obtained from enzymatic hydrolysis are generally recognized as safe (GRAS) [8]. Pumpkin seed proteins (PSPs) (*Cucurbita pepo*) are gaining increasing attention due to their nutritional profile and potential for generating bioactive peptides, which can be explored in various sectors, including pharmaceutical and food industries [9]. However, the structural complexity of PSP makes them less soluble compared to animal proteins, which consequently hinders the enzyme access to peptide bonds, resulting in a low peptide yield and reduced bioactivity [9]. In addition to these challenges, enzymatic processes require mild temperatures (40~50 °C) and extended processing times (6~8 h) [9]. In this context, developing strategies to enhance the efficiency of these processes is an important issue in the field of food science and technology.

The ultrasound (US) has been investigated as a strategy to improve the enzymatic hydrolysis of plant proteins, with studies reporting its potential to enhance process efficiency, peptide yield, and cost-effectiveness [3,7,9]. Our research group has strategically investigated PSP hydrolysis through the action of Brauzyn^®^, Flavourzyme^®^, and Neutrase^®^, employing the US in the following two approaches: (i) the pretreatment of enzymes [9] and (ii) the pretreatment of PSP [3]. Our findings indicate that sonication, applied exclusively to enzymes, induced conformational changes, enhancing enzymatic activity, while PSP sonication prior to hydrolysis led to significant modifications in the protein structure, resulting in up to 100% and 89% increases in the reaction rate and degree of hydrolysis (DH), respectively. Among these pre-hydrolysis treatments, substrate sonication appears more advantageous than enzyme pretreatment, as a DH optimization suggests a greater production of low molecular weight peptides, potentially enhancing the bioactivity of the hydrolysates [3]. However, US-assisted PSP hydrolysis has not yet been fully elucidated. The literature suggests that the simultaneous application of sonication to both substrates and enzymes during the reaction can modify their structures through mechanical and cavitation effects, potentially enhancing process efficiency and the production of bioactive peptides [7,10]. Thus, this study aims to assess whether this combined approach leads to a greater PSP hydrolysis compared to other methods and whether the resulting peptides exhibit superior functional properties.

The choice of enzymes is a key factor in producing hydrolysates with enhanced multifunctional properties [9]. Although studies have evaluated the effect of an US on the assisted proteolysis of various protein substrates [11,12,13], none have performed a comparative analysis of the use of commercial enzymes with different costs and specificities. This gap makes it challenging to determine the best strategy for producing hydrolysates of industrial interest from the same protein source. Furthermore, the efficiency of US-assisted processes can vary according to factors such as time and temperature [14], making it essential to identify the conditions that require lower energy consumption, which could positively impact the production costs of bioactive peptides. 

Thus, by evaluating two process parameters (time and temperature of the catalysis, with and without US) and three biocatalysts (Brauzyn^®^, Flavourzyme^®^, and Neutrase^®^), this study addresses these knowledge gaps and aims to identify the optimal conditions for maximizing the positive effects of US-assisted PSP hydrolysis. Additionally, this work investigates the kinetics of US-assisted PSP hydrolysis and its consequences on the macrostructural characteristics, solubility, and antioxidant activity of the produced peptides.

## 2. Materials and Methods

### 2.1. Enzymes and Pumpkin Seed Protein (PSP)

Three commercial enzymes were utilized in this study: Brauzyn^®^ (papain, 90 TUP/mg) provided by Prozyn Biosolutions (São Paulo, Brazil); Flavourzyme^®^ (*Bacillus oryzae*, ≥500 U/g) and Neutrase^®^ (*Bacillus amyloliquefaciens* endoprotease, 0.8 U/g) supplied by Novozymes Latino Americana Ltda. (Araucária, Brazil).

The pumpkin seeds (with 29.8% moisture, 21.9% protein, and 48.3% non-protein solids–determined by the authors) were kindly provided by Doces Mirahy (Miraí, MG, Brazil). Pumpkin seed proteins (PSPs) (1.8% moisture, 78.6% protein, and 19.7% non-protein dry extract, composed mainly of minerals and lipid and carbohydrate residues) were obtained by alkaline extraction followed by isoelectric precipitation, as described in the study by Pacheco et al. [3].

**Figure 1:**
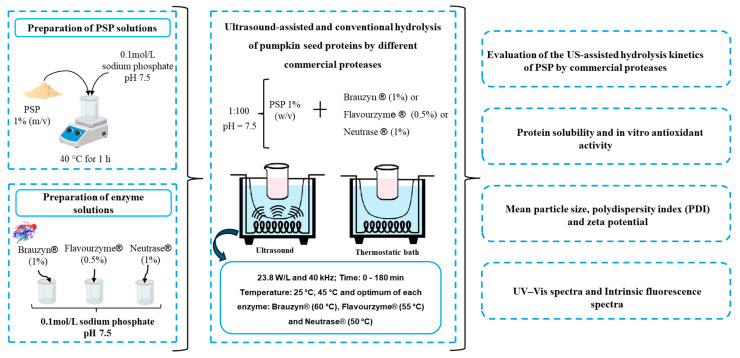
Flowchart of experiments evaluating US-assisted hydrolysis of PSPs.

Subsequently, PSPs were subjected to a US-assisted enzymatic hydrolysis, and the peptides obtained were evaluated for macrostructural characteristics, solubility, and in vitro antioxidant activity (Figure 1).

### 2.2. PSP Hydrolysis by Commercial Proteases Under US-Assisted Reaction

For the hydrolysis assays, PSPs were dispersed in 0.1 mol/L sodium phosphate (Synth, São Paulo, Brazil) buffer (pH 7.5) at a concentration of 1% (*w*/*v*), based on the Michaelis–Menten constant (Km) values defined in the previous study Pacheco et al. [9]. The mixture was then stirred at 40 °C for 1 h. The enzyme solutions were prepared in the same buffer (pH 7.5), with concentrations of 1% for Brauzyn^®^ and Neutrase^®^ and 0.5% for Flavourzyme^®^, resulting in final activities of 219.6 U/mL, 214.3 U/mL, and 220.8 U/mL on PSP, respectively. These concentrations were selected based on the conditions studied in a previous work by our research group to ensure an adequate comparison of the results obtained [3].

The US-assisted hydrolysis of PSPs was conducted in an ultrasonic bath (Unique Inc., model USC 2800 A, Indaiatuba, Brazil), operating at a frequency of 40 kHz, with a nominal power of 450 W and a volumetric power of 23.8 W/L, measured according to the calorimetric method described by O’Donnell et al. [15]. A stainless-steel heat exchanger was used to maintain the process temperatures, with water recirculation using an external ultra-thermostatic bath.

To perform the processes, 50 mL of the PSP solution was placed in a glass beaker inside the ultrasonic bath at the highest ultrasonic intensity position [16]. The enzyme solution (prepared as previously described) was added to this beaker in an enzyme/substrate ratio of 1:100 [9]. Sonication was performed for 180 min of hydrolysis at 25 °C, 40 °C, and at the optimal temperature condition, for each enzyme studied (60 °C for Brauzyn^®^, 55 °C for Flavourzyme^®^, and 50 °C for Neutrase^®^) [9]. Additionally, hydrolyses under the same conditions of temperature, pH, enzyme, and substrate concentration were performed in a thermostatic bath to produce control samples (conventional hydrolysis).

### 2.3. Evaluation of PSP Hydrolysis

#### 2.3.1. Degree of Hydrolysis (DH)

The degree of hydrolysis (DH) was determined using the pH-stat method, as described by Santos et al. [3] over a period of 180 min. The initial pH was adjusted to 7.5 and maintained at this value by adding NaOH (Synth, São Paulo, Brazil) (0.1 mol/L) at 0, 5, 15, 30, 45, 60, 90, 120, and 180 min of hydrolysis. At these time points, the DH was measured according to Equation (1).

(1) $${DH}_{t}=\frac{h}{h_{tot}}*100=\frac{V*C}{\alpha *M*h_{tot}}*100$$

where V (mL) denotes the volume of NaOH used, C (mol/L) is the NaOH concentration, α represents the average dissociation degree of the α-NH_2_ groups released during hydrolysis at a given temperature and pH (0.414 for all enzymes, as calculated by Kurozawa et al. [17]), M (g) is the protein mass to be hydrolyzed in the mixture, h_tot_ (mmol/g) indicates the maximum molar number of peptide groups per unit of protein mass, and h is the molar number of peptide groups obtained after t minutes of hydrolysis per unit of protein mass.

#### 2.3.2. Modeling of Hydrolysis Kinetics

The modeling of the hydrolysis kinetics of PSPs using three different enzymes was evaluated using a first-order model, where the reaction rate was demonstrated by the increase in DH, according to Equation (2).

(2) $${DH}_{t}={DH}_{\infty }\left(1-e^{-kt}\right)$$

where DH_t_ represents the degree of hydrolysis (%) at time t; DH∞ is the final degree of hydrolysis (%); t is the reaction time (min); and k is the hydrolysis rate constant (min^−1^), which indicates the speed of the hydrolysis reaction at a constant temperature.

### 2.4. Macrostructure, Solubility, and Antioxidant Activity of the Produced Hydrolysates

To evaluate the hydrolysates, after 45 and 180 min of US-assisted hydrolysis, performed as described in Section 2.2, at the optimum temperature for each enzyme, 15 mL aliquots were collected and boiled for 10 min to stop the reactions. Then, the hydrolysates obtained were analyzed for protein solubility (Section 2.4.1), in vitro antioxidant activity (Section 2.4.1), and macrostructural characteristics (Section 2.4.2 and Section 2.4.3).

#### 2.4.1. Evaluation of Protein Solubility and In Vitro Antioxidant Activity

The native protein (non-hydrolyzed) and the hydrolysates obtained were centrifuged at 7500× *g* for 15 min at 4 °C. The supernatant obtained from each sample was used to determine the protein solubility at different pH values (2, 4, 6, 8, and 10) and in vitro antioxidant activity, according to methodologies described by Magalhães et al. [14].

For the DPPH radical scavenging activity, the hydrolysate was prepared by dispersing it in 0.1 M sodium phosphate buffer (pH 7.5) at a concentration of 1 mg/mL. An aliquot of 700 μL of the prepared sample was mixed with 700 μL of ethanol (99.5%) (Dinâmica, São Paulo, Brazil), and 175 μL of ethanol containing 0.01% DPPH (Sigma-Aldrich Brazil Ltda., São Paulo, Brazil) were added into a test tube. The mixture was kept at room temperature in the dark for 60 min, and its absorbance was measured at 517 nm. The sample was blanked with 0.1 M sodium phosphate buffer (pH 7.5). The DPPH radical scavenging capacity was expressed as a percentage of inhibition and was calculated using Equation (3).

(3) $$Inhibition\;(\%)=\frac{{Abs}_{0\;min}-{Abs}_{60\;min}}{{Abs}_{0\;min}}\times 100$$

For the ABTS radical scavenging activity, a stock solution containing ABTS (7 mM) and potassium persulfate (2.45 mM) (1:1 ratio) was prepared and stored in the dark at 4 °C for 12–16 h before use. The solution was diluted to an absorbance of 0.700 ± 0.02 at 734 nm. Then, 150 μL of the diluted hydrolysate (1:100) was mixed with 2.85 mL of the ABTS (Sigma-Aldrich Brazil Ltda., São Paulo, Brazil) solution. After 60 min in the dark, the absorbance was measured at 734 nm, and the activity was calculated by Equation (3).

#### 2.4.2. Mean Particle Size, Polydispersity Index (PDI) and Zeta Potential

The mean particle size, polydispersity index (PDI), and zeta potential of the PSPd (native protein–non-hydrolyzed) and hydrolysates obtained by conventional and US-assisted hydrolysis were measured at 25 °C using a Zetasizer Nano ZS (Malvern Instruments, United Kingdom). For the analysis, native PSPs and the hydrolysates obtained were diluted in 0.1 mol/L phosphate buffer (pH 7.5) at a ratio of 1:100. Then, each system was transferred to a cuvette and analyzed at 25.0 ± 0.1 °C according to the methodology described by Pacheco et al. [3].

#### 2.4.3. UV–Vis Spectra and Intrinsic Fluorescence Spectra

The UV-Vis absorbance and intrinsic fluorescence spectra of the hydrolysates were determined using a SpectraMax M5 fluorescence spectrophotometer (Molecular Devices, San Diego, CA, USA). For each measurement, 300 μL of the hydrolysates were centrifuged at 7500× *g* for 15 min at 4 °C, and the supernatant was transferred to a 96-well plate at room temperature (25 ± 1 °C). The UV-Vis absorbance spectra were recorded in the range of 200–400 nm [7], while for intrinsic fluorescence, the samples were excited at the excitation wavelength of tryptophan (295 nm), and the emission spectra were recorded in the wavelength range of 310–450 nm [7].

### 2.5. Experimental Design and Statistical Analysis

Protein hydrolysis was performed in three repetitions, and the analytical evaluations of the hydrolysates were carried out in triplicate, totaling 9 measurements for each process condition evaluated (n = 9). Results were expressed as mean ± standard deviation. The means of protein solubility, in vitro antioxidant activity, mean particle size, PDI, and zeta potential analyses were analyzed using one-way ANOVA, followed by Tukey’s post hoc test for comparisons between samples with 95% confidence (Statistical Analysis System—SAS Institute, Cary, NC, USA; version 9.2). Model parameters for obtaining k and DH∞ were determined by nonlinear regression using Curve Expert Professional software (version 2.6.5, Hyams Development, Chattanooga, TN, USA) with a significance level set at 95%.

## 3. Results and Discussion

### 3.1. PSP Hydrolysis by Commercial Proteases Under US-Assisted Reaction

DH indicates the level of cleavage of peptide bonds in a protein and is usually used to compare hydrolysates [7]. In the present study, the investigation of DH is particularly important because the proteases studied have distinct specificities. Each enzyme acts on specific cleavage sites within the protein structure, releasing different hydrolysates [18].

**Figure 2:**
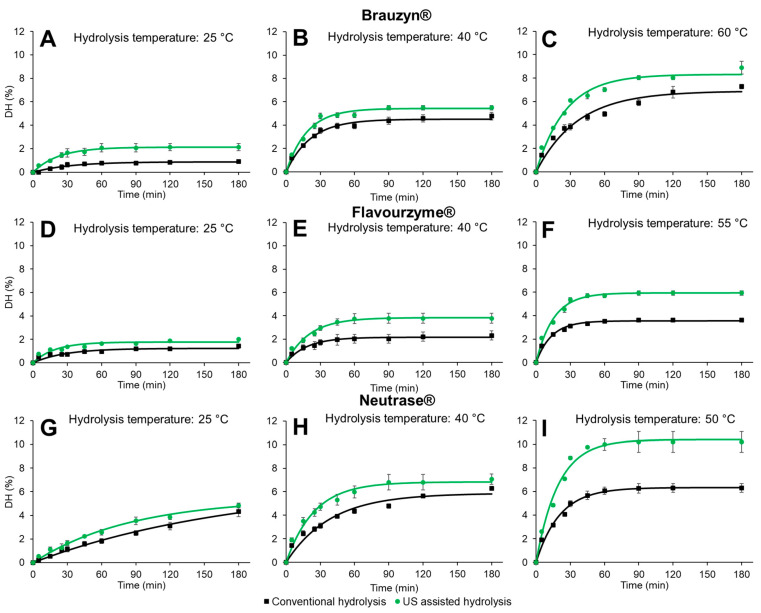
Conventional and ultrasound-assisted hydrolysis of the pumpkin seed protein (PSP) using Brauzyn^®^ (**A**–**C**), Flavourzyme^®^ (**D**–**F**), and Neutrase^®^ (**G**–**I**) at different temperatures. The dots are the experimental values, the vertical bars are the standard deviation for each condition, and the curves (continuous lines) are the adjusted model of Equation (2). DH = Degree of hydrolysis (%).

The DH curves of the PSP, obtained using the enzymes Brauzyn^®^ (A, B, and C), Flavourzyme^®^ (D, E, and F), and Neutrase^®^ (G, H, and I), in processes with or without US assistance, are shown in Figure 2. 

The curve profiles demonstrated that proteolysis was faster and, especially at higher temperatures, reached higher degrees of hydrolysis in the US-assisted processes. Furthermore, the kinetic parameters, namely the final degree of hydrolysis (DH∞) and the hydrolysis rate (k parameter), determined through Equation (2) (Table 1), provided significant insights into the efficiency of the enzymatic processes.

When comparing the conventional and US-assisted hydrolysis processes under the same conditions (enzyme and temperature), the k results revealed that the US-assisted reactions had similar hydrolysis rates, except when Flavourzyme^®^ was used at 25 °C and/or when Neutrase^®^ was used at 40 °C (*p* ≤ 0.05). Under these conditions, the US-assisted process increased the hydrolysis rate by 47.2% and 57.7%, respectively. Furthermore, sonication during proteolysis enhanced the production of hydrolysates, increasing the DH value in most of the conditions tested (*p* ≤ 0.05), demonstrating that the US did not negatively affect the activity of commercial enzymes under the evaluated conditions. 

As expected, for most of the evaluated conditions, increasing the temperature led to an increase in DH∞ (Table 1), reaching the maximum DH∞ at the optimum temperature of each enzyme. Furthermore, after 45 min (plateau point for most of the curves) and 180 min of reaction, the degree of hydrolysis (DH_45min_ and DH_180min_) of the US-assisted reactions was higher compared to the respective DHs of the conventional reactions under optimum conditions (Table 1—*p* ≤ 0.05). These results corroborate the findings from other studies reporting that the US can enhance enzymatic reactions [7,14,19,20,21].

A comparative evaluation of the DH∞ results shows that the US was more effective in enhancing hydrolysis by Neutrase^®^, followed by Flavourzyme^®^ and Brauzyn^®^ (maximum increases of 65.1%, 63.9%, and 20.3%, respectively) (*p* ≤ 0.05). The increase in the degree of hydrolysis by US is related to the following three factors that can occur simultaneously in the reaction medium: (i) modifications in the substrate; (ii) structural changes in the enzymes; and (iii) increased mass transfer [22,23]. These changes occur at the microscopic level and are caused by the physical effects of the US, such as cavitation, shear forces, and shock waves [24], which rupture cross-links and lead to the exposure of internal protein groups, optimizing the enzyme–substrate interaction [11]. At the same time, the US is capable of breaking hydrogen bonds and van der Waals interactions in enzymes, altering their conformational structures and, consequently, potentially making the enzymes more active due to the spatial exposure of active sites and regions important for catalysis [24]. Furthermore, mass transfer is favored in US assisted hydrolysis, as the intense energy release during the acoustic cavitation reduces the diffusion barrier between enzyme and substrate, accelerating the reaction rate [14]. 

In this perspective, it is assumed that these effects, in a synergistic or additive manner, were fundamental to achieving the DH∞ values observed in US-assisted reactions under optimal conditions (DH∞ of 8.3%, 5.9%, and 10.4% after hydrolysis by Brauzyn^®^, Flavourzyme^®^, and Neutrase^®^, respectively). According to the findings of Vioque et al. [25], the hydrolysates with DH lower than 8% are used as functional ingredients and flavorings in foods due to improved solubility, emulsifying, and foaming properties, while those with DH above 8% possess bioactive potential (e.g., antioxidant activity), making them relevant for the nutraceutical industry [25,26]. Therefore, our findings indicate that the US was able to enhance PSP cleavage, producing peptides with functional potential, especially for Flavourzyme^®^, and bioactive peptides for Brauzyn^®^ and Neutrase^®^. 

It is worth noting that the DH∞ results of the US-assisted Flavourzyme^®^- and Neutrase^®^-catalyzed reactions at 40 °C are promising, as they were similar to those of the conventional reaction at the optimal temperature of these enzymes (55 and 50 °C, respectively) (*p* ≤ 0.05). From an industrial perspective, these results are important, as they allow the production of relevant peptides with lower energy consumption [27]. Additionally, the US is considered a non-toxic, environmentally friendly technology, that does not require high installation and maintenance costs compared to other physical processing technologies, thereby offsetting its initial investment [7].

### 3.2. Evaluation of Protein Solubility

Protein solubility is a physicochemical property that governs the functionality of proteins, dictating the applicability of these macromolecules in food systems such as foams, gels, and emulsions [7,28]. Table 2 presents the solubility results as a function of pH (2 to 10) for native PSP and hydrolysates obtained after 45 and 180 min of hydrolysis, both with and without US intervention. 

As expected, the native PSPs showed lower solubility values at pH 4.0 and 6.0 (14% and 12%, respectively) (*p* ≤ 0.05), given its proximity to pH 5.0, which is its isoelectric point (pI) [7,29]. In contrast, at pH values further from the pI, the solubility of native PSP increased (*p* ≤ 0.05), reaching a maximum solvation of 81% at pH 10. Similar results were also found in other studies [3,9,30].

As shown in Table 2, an enzymatic hydrolysis with the three enzymes increased PSP solubility for most of the evaluated conditions, compared to the non-hydrolyzed protein (native PSP) (*p* ≤ 0.05), especially after 180 min, when Brauzyn^®^ and Neutrase^®^ were used as biocatalysts. An enzymatic hydrolysis has been effectively used to improve the solubility and functional properties of PSPs due to the breakdown of the polypeptide chain of proteins, with the release of smaller peptides and subsequent exposure of positively or negatively charged groups (mainly amine and carbonyl) to the solvent [31]. These effects are more evident in the regions close to the isoelectric point of PSPs (between pH 4.0 and 6.0) and are crucial to improving the applications of hydrolysates in foods with pH within this range.

An US-assisted hydrolysis improved the hydrolysate solubility (6.6–100%) compared to that obtained through conventional hydrolysis (*p* ≤ 0.05), particularly at pH 4.0–8.0 and, in some conditions, at pH 2.0. The magnitude of these differences aligns with the DH results (Table 1), suggesting that US-assisted reactions were more effective in producing potentially bioactive peptides and, consequently, more soluble peptides. This enhances the potential applications of hydrolysates as ingredients in the food industry [9,32]. 

### 3.3. Evaluation of In Vitro Antioxidant Activity 

Antioxidant compounds derived from plant by-products have gained relevance in recent research, with special emphasis on bioactive peptides [33]. In this study, the antioxidant potential was measured by in vitro ABTS and DPPH radical scavenging tests (Table 3). A conventional enzymatic hydrolysis, after 180 min, significantly increased the antioxidant activity in all cases, as demonstrated by both methods (*p* ≤ 0.05). Specifically, compared to the native protein (non-hydrolyzed), increases of 516.7%, 250%, and 366.7% were observed for the DPPH radical, for Brauzyn^®^, Flavourzyme^®^, and Neutrase^®^, respectively. For the ABTS radical, the increases were 262.5%, 193.8%, and 162.5%, respectively.

In the US-assisted reactions, all hydrolysates showed a greater capacity to inhibit DPPH and ABTS radicals, compared to conventional reactions (*p* ≤ 0.05), with particular emphasis on those obtained using Flavourzyme^®^, which exhibited increases of 52.4% and 42.6% in DPPH and ABTS radical inhibition, respectively, compared to their counterparts produced by conventional hydrolysis after 180 min (*p* ≤ 0.05). These results highlight the potential of combining the US with enzymatic hydrolysis to enhance the antioxidant properties of protein hydrolysates. The increase in antioxidant activity observed in peptides obtained without sonication can be attributed to enzymatic hydrolysis, which facilitates the release of bioactive peptides from native proteins [34]. In contrast, the combination of sonication with enzymatic hydrolysis promotes the unfolding of protein structures, increasing enzyme accessibility to the binding sites and consequently enhancing the release of antioxidant peptides capable of acting as electron donors [11], in line with the DH data.

These findings are consistent with the previous studies on US-assisted enzymatic hydrolysis. Quan et al. [13] reported that hydrolysates from oyster proteins exhibited a 66.8% and 55.2% increase in antioxidant activity for DPPH and ABTS radicals, respectively, after 4 h of hydrolysis. Similarly, Liu et al. [35] observed increases of 73.2% and 44.9% in antioxidant activity for DPPH and ABTS radicals, respectively, in whey protein hydrolysates after 60 min of hydrolysis. In this context, the antioxidant activity observed in the present study can be attributed to peptides with a high content of hydrophobic and aromatic amino acids, such as Trp, Tyr, Pro, and Phe, which have been reported to contribute to radical scavenging due to their phenolic groups acting as hydrogen donors [9]. Furthermore, peptides with Pro, Val, Phe, and His residues at the N- and C-terminal positions may enhance antioxidant capacity by improving interactions with fatty acids and increasing their ability to capture lipid free radicals [14].

### 3.4. Mean Particle Size, Polydispersity Index (PDI) and Zeta Potential

The results of mean particle size, polydispersity index, and zeta potential at pH 7.5 of the peptides obtained by conventional and US-assisted hydrolysis can be seen in Table 4. The hydrolysates obtained through conventional reactions by the action of Brauzyn^®^, Flavourzyme^®^, and Neutrase^®^ exhibited particle sizes of 566 nm, 377 nm, and 513 nm after 45 min of hydrolysis, respectively. Significant reductions (*p* ≤ 0.05) in particle size were observed during hydrolysis, with decreases of up to 46.6%, 55.4%, and 50.3% for Brauzyn^®^, Flavourzyme^®^, and Neutrase^®^, respectively, between the 45 min and 180 min time points (Table 4).

Studies have shown that sonication, through mechanical forces, disrupts intermolecular interactions in peptides and enhances the catalytic activity of enzymes, thereby facilitating the formation of smaller fragments [36,37]. This effect was also observed in the present study, where significant reductions in particle size (*p* ≤ 0.05) were noted after 45 min of US-assisted hydrolysis for all enzymes. Furthermore, after 180 min, US-assisted hydrolysis using Brauzyn^®^ and Flavourzyme^®^ resulted in particles approximately 26.2% smaller (*p* ≤ 0.05).

The PDI values presented in Table 4 serve as a metric for assessing the homogeneity of the particles formed, with lower values indicating more homogeneous particles and improved dispersibility in the samples [38]. In general, hydrolysis promoted greater particle homogeneity, as evidenced by a significant reduction in PDI between 45 and 180 min of hydrolysis. The greatest reductions during conventional hydrolysis were observed for Brauzyn^®^ (29.4%), followed by Neutrase^®^ (27.6%) and Flavourzyme^®^ (19.6%) (Table 3). The US-assisted hydrolysis further enhanced this effect. After 45 min of hydrolysis, all samples produced via US-assisted reactions exhibited lower PDI values compared to conventional reactions, with reductions of 13.1%, 16.0%, and 18.5% for Brauzyn^®^, Flavourzyme^®^, and Neutrase^®^, respectively (*p* ≤ 0.05). However, after 180 min, only the Brauzyn^®^ hydrolysate maintained significantly lower PDI values (20.3% reduction) in the US-assisted reaction compared to the conventional process (*p* ≤ 0.05). This result may be attributed to the higher hydrolysis rate facilitated by US-assisted reactions, which likely accelerated the particle size reduction and improved the homogeneity in a shorter time. Notably, this suggests that US-assisted hydrolysis could achieve the desired level of particle homogeneity within 45 min, offering significant advantages for industrial applications. Shorter hydrolysis times translate to a reduced energy consumption and, consequently, lower operating costs, highlighting the process’s potential for improving efficiency in industrial settings. 

These findings align with the observed increase in protein solubility (Table 2), demonstrating that the particle size reduction promoted by enzymatic hydrolysis can enhance techno-functional properties. Similarly, the US-assisted enzymatic hydrolysis has been shown to improve the solubility of goat milk casein, which can be attributed to the reduced molecular weight of the hydrolysates and their higher solvation capacity [14]. In parallel, previous studies have reported that an ultrasound treatment reduces the particle size and PDI of pea protein [39] and album seed protein [40], leading to the formation of more uniformly dispersed particles (*p* < 0.05) [9]. From a technological perspective, these characteristics are crucial for improving food product stability, such as protein-based beverages. Additionally, in pharmaceutical applications, reducing particle size can enhance bioavailability by increasing absorption and enabling the controlled release of drugs [3].

Zeta potential is a measure of the surface charge on the particles in a solution, with higher values generally indicating a more stable system due to reduced particle aggregation [37]. The zeta potential values of the peptides obtained after 180 min of conventional hydrolysis were −49.8 mV (Brauzyn^®^), −58.8 mV (Flavourzyme^®^), and −55.4 mV (Neutrase^®^), suggesting that the hydrolysis with Flavourzyme^®^ produced peptides with more repulsive surface charges. Additionally, the hydrolysis performed under sonication favored the generation of peptides with more repulsive zeta potentials, compared to conventional hydrolysis at both 45 and 180 min (*p* ≤ 0.05). Specifically, increases in repulsivity of 44.5% (Brauzyn^®^), 10.5% (Flavourzyme^®^), and 17.4% (Neutrase^®^) were observed after 45 min of US-assisted hydrolysis, and increases of 21.4% (Brauzyn^®^), 6.1% (Flavourzyme^®^), and 11.3% (Neutrase^®^) were noted after 180 min. These results indicate that the ultrasound promoted structural changes in the hydrolysates, enhancing the stability and dispersion of the particles. These findings align with the results of the mean particle size analysis.

### 3.5. UV–Vis Spectra and Intrinsic Fluorescence Spectra

**Figure 3:**
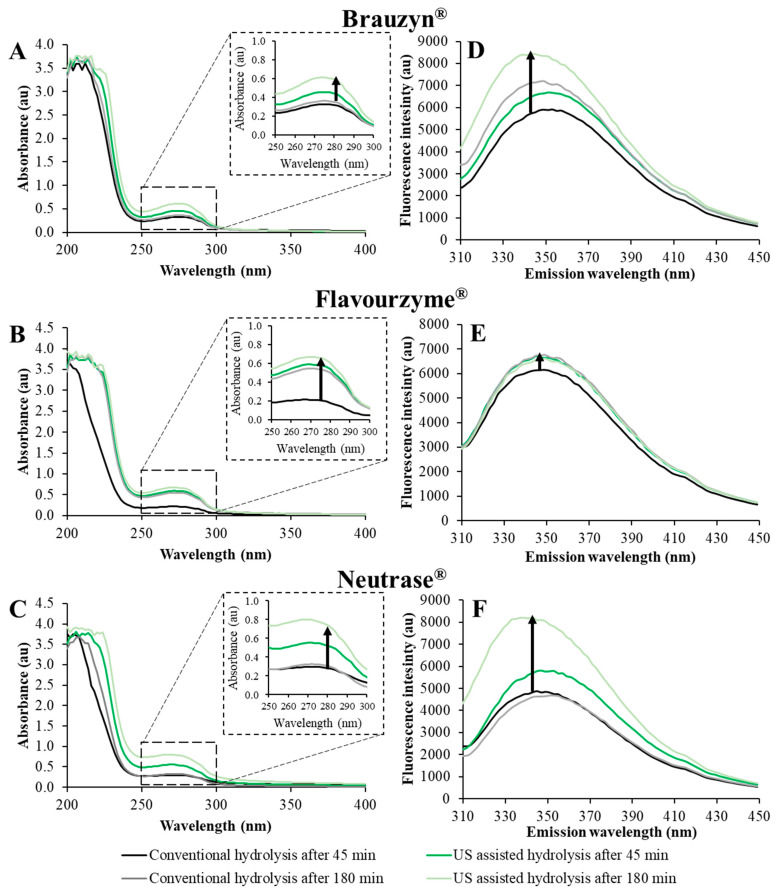
UV–Vis spectra (**A**–**C**) and Intrinsic fluorescence spectra (**D**–**F**) at pH 7.5 for protein hydrolysates obtained after 45 and 180 min of conventional and ultrasound-assisted hydrolysis.

UV-Vis spectroscopy and intrinsic fluorescence are sensitive methods for identifying modifications in the tertiary structure of proteins or peptides, with a particular emphasis on aromatic amino acids such as tryptophan and tyrosine [7,41]. During conventional hydrolysis for 180 min under the action of Brauzyn^®^, Flavourzyme^®^, and Neutrase^®^, the peptides presented maximum absorbance peaks at 280 nm of 0.354, 0.523, and 0.310, respectively (Figure 3A–C and Table 5). 

For US-assisted hydrolysis, significant increases in the UV absorbance intensity of the peptides obtained, compared to the conventional reaction, were observed after 45 min and 180 min of hydrolysis (highlighted by black arrows in Figure 3A–C) (*p* ≤ 0.05) (Table 5). The highest UV absorbance intensity measured at 280 nm, after 180 min of US-assisted hydrolysis of PSP, was 0.742 for Neutrase^®^, 0.638 for Flavourzyme^®^, and 0.595 for Brauzyn^®^, indicating increases of 139.3%, 22%, and 68.1%, respectively, compared to the conventional reaction (*p* ≤ 0.05). This result can be attributed to the higher degree of hydrolysis in the US-assisted reactions, which contributed to an increase in the content of hydrophobic peptides, as well as to the cavitation effects and mechanical forces generated by sonication, which favored molecular unfolding and the exposure of additional hydrophobic groups [41].

Regarding the intrinsic fluorescence intensity (Figure 3D–F and Table 5), the spectra of the peptides obtained through conventional hydrolysis with Brauzyn^®^, Flavourzyme^®^, and Neutrase^®^ presented a single peak, with the maximum fluorescence wavelength (λmax) of 353, 349, and 351 nm, respectively, after 180 min of hydrolysis. In general, the increase in hydrolysis time led to a higher maximum intrinsic fluorescence intensity (highlighted by black arrows in Figure 3D–F), likely due to an increased exposure to aromatic amino acids, such as tryptophan and tyrosine [37]. Comparatively, it was observed that the US-assisted hydrolysis induced changes in the fluorescence spectra of the obtained peptides, particularly by the action of the enzymes Brauzyn^®^ and Neutrase^®^. Notably, a shift in λmax to 343 nm was observed in both cases, accompanied by increases in the peak intensities of 19.9% for Brauzyn^®^ and 75.6% for Neutrase^®^, compared to conventional hydrolysis after 180 min (Table 4—*p* ≤ 0.05).

### 3.6. Comparative Study Between Ultrasound Treatments for Each Enzyme

The results presented in Table 6 show the percentage gains obtained for each enzyme under different US treatment conditions, considering the parameters of the degree of hydrolysis (DH∞), soluble protein at pH 4 and 6, and antioxidant activity (DPPH and ABTS) in the reactions conducted at the optimum temperature for each enzyme. The data from the present study (US-assisted PSP hydrolysis) were compared with those from the previous studies conducted by our research group, where PSP hydrolysis was performed after a prior sonication of the enzymes [9] or the substrate [3], without the application of ultrasound during the enzymatic reaction. 

The different US treatments (enzyme pretreatment, substrate pretreatment, and assisted treatment) had distinct impacts on the gains of the evaluated attributes, highlighting that the type of process approach directly influences the performance of each enzyme and, consequently, the characteristics of the hydrolysates. Therefore, the choice of the best treatment depends on the desired properties of the hydrolysates and the enzyme used. For instance, when a higher degree of hydrolysis is desired, the best strategy is the ultrasonic pretreatment of the substrate, when using the enzymes Brauzyn^®^ and Neutrase^®^ (gains of 32.4% and 89.1%, respectively—Table 6). However, for the use of Flavourzyme^®^, the optimal strategy is to conduct a US-assisted reaction (gain of 63.9%).

For gains in soluble proteins, the best strategy when using Flavourzyme^®^ is to perform either enzyme pretreatment or the assisted reaction. In contrast, for the other enzymes, both ultrasonic approaches result in similar gains (Table 6). Regarding the antioxidant activity, specifically for the DPPH radical, the greatest gains for Flavourzyme^®^ were observed with substrate pretreatment and in the assisted reaction. However, for the ABTS radical, enzyme pretreatment (Flavourzyme^®^ and Neutrase^®^) was the most effective approach (Table 6). Overall, the US-assisted reaction emerged as the most efficient strategy, particularly for Flavourzyme^®^. This approach yielded a high degree of hydrolysis (DH∞ of 63.9%) and a high protein solubility at pH 6 (100%), along with notable antioxidant activity, particularly for DPPH after 45 min of hydrolysis (86.7%). These results suggest that the US-assisted reaction not only enhances the individual effects on the substrate and the enzyme, but also accelerates the mass transfer during the reactions. This improves the enzyme access to active sites, maximizing catalytic efficiency and facilitating the release of potentially functional and bioactive peptides.

It is important to emphasize that the final decision regarding the strategy will depend on the operational conditions and costs involved in the process. For instance, enzyme sonication requires equipment with significantly lower processing capacity in liters per hour compared to substrate processing or assisted hydrolysis. However, it necessitates the immediate use of the enzyme after sonication, as the structural changes observed may be reversible, directly impacting its catalytic activity. In the case of substrate processing, the changes are believed to be more stable, not requiring immediate processing after sonication. Finally, the assisted hydrolysis tends to be more complex, but it ensures that the protein structures will not revert, as the interval between pre-processing and hydrolysis is eliminated (unlike in the other approaches) and, in general, results in greater improvements in the properties of the hydrolysates produced.

Therefore, this study represents one of the few that enables a comprehensive comparison of the various methods for incorporating sonication in the production of protein hydrolysates via enzymatic hydrolysis. This is particularly relevant as the conditions (pH, temperature, dilution, process time, raw material preparation, etc.) were consistently reproduced in this study and in the studies previously conducted by the group [3,9], using three commercially significant enzymes. Consequently, these data are crucial for advancing the scientific knowledge in this field and, more importantly, for the industrial applicability of the process.

## 4. Conclusions

The application of ultrasonic technology in combination with enzymatic hydrolysis proved highly effective in enhancing the functional and bioactive properties of protein hydrolysates derived from PSPs. The results indicated that the use of the ultrasound led to a significant increase in DH, thereby improving the efficiency of the Brauzyn^®^, Flavourzyme^®^, and Neutrase^®^ enzymes. This enhanced efficiency was attributed to acoustic cavitation, which induced structural modifications in both the substrate and the enzymes, thereby improving enzyme–substrate interactions and facilitating mass transfer. Furthermore, the hydrolysates exhibited improved antioxidant properties and enhanced solubility across a wide pH range, making them suitable for potential applications in the food and nutraceutical industries. The hydrolysates obtained through the ultrasound-assisted reactions also displayed smaller particle sizes, increased colloidal stability, and higher intrinsic fluorescence intensities. These findings suggest that the industrial viability of ultrasound technology lies in it enabling the production of functional hydrolysates at lower temperatures and reduced processing times, contributing to energy efficiency and potential cost savings. Thus, the integration of the ultrasound in enzymatic hydrolysis represents a promising, sustainable, and economically advantageous approach for producing functional and bioactive protein ingredients from pumpkin seeds.

## Figures and Tables

**Table 1:** Parameters of Equation (2) adjusted to conventional and ultrasound-assisted hydrolysis of pumpkin seed protein using proteases at different temperatures.

Enzyme	Hydrolysis	T (°C)	k (min^−1^)	DH∞ (%)	R^2^	DH_45 min_ (%)	DH_180 min_ (%)
Brauzyn^®^	Conventional hydrolysis	25	0.031 ± 0.012 ^cd^	0.9 ± 0.0 ^f^	0.966	0.7 ± 0.1 ^e^	0.9 ± 0.1 ^e^
		40	0.047 ± 0.003 ^ab^	4.5 ± 0.3 ^d^	0.994	3.9 ± 0.2 ^c^	4.8 ± 0.3 ^c^
		60	0.027 ± 0.001 ^d^	6.9 ± 0.3 ^b^	0.982	4.7 ± 0.3 ^b^	7.3 ± 0.2 ^b^
	US assisted hydrolysis	25	0.045 ± 0.003 ^abc^	2.1 ± 0.3 ^e^	0.991	1.7 ± 0.3 ^d^	2.1 ± 0.3 ^d^
		40	0.055 ± 0.003 ^a^	5.4 ± 0.2 ^c^	0.993	4.9 ± 0.2 ^b^	5.5 ± 0.2 ^c^
		60	0.039 ± 0.003 ^bcd^	8.3 ± 0.4 ^a^	0.991	6.5 ± 0.3 ^a^	8.9 ± 0.5 ^a^
Flavourzyme^®^	Conventional hydrolysis	25	0.036 ± 0.000 ^d^	1.2 ± 0.0 ^d^	0.937	0.9 ± 0.0 ^d^	1.4 ± 0.0 ^d^
		40	0.054 ± 0.008 ^bc^	2.2 ± 0.4 ^c^	0.984	2.0 ± 0.5 ^c^	2.3 ± 0.4 ^c^
		55	0.074 ± 0.006 ^a^	3.6 ± 0.1 ^b^	0.993	3.3 ± 0.1 ^b^	3.6 ± 0.1 ^b^
	US assisted hydrolysis	25	0.053 ± 0.000 ^c^	1.8 ± 0.0 ^cd^	0.949	1.4 ± 0.0 ^cd^	2.0 ± 0.0 ^cd^
		40	0.049 ± 0.005 ^c^	3.8 ± 0.4 ^b^	0.992	3.5 ± 0.3 ^b^	3.8 ± 0.4 ^b^
		55	0.066 ± 0.002 ^ab^	5.9 ± 0.2 ^a^	0.994	5.7 ± 0.2 ^a^	5.9 ± 0.2 ^a^
Neutrase^®^	Conventional hydrolysis	25	0.006 ± 0.001 ^d^	6.7 ± 0.2 ^bc^	0.996	1.6 ± 0.2 ^d^	4.3 ± 0.4 ^c^
		40	0.026 ± 0.002 ^c^	5.9 ± 0.1 ^bc^	0.977	3.9 ± 0.2 ^c^	6.3 ± 0.2 ^b^
		50	0.048 ± 0.003 ^ab^	6.3 ± 0.4 ^bc^	0.994	5.7 ± 0.4 ^b^	6.3 ± 0.4^b^
	US assisted hydrolysis	25	0.012 ± 0.002 ^d^	5.4 ± 0.4 ^c^	0.994	2.2 ± 0.1 ^d^	4.8 ± 0.2 ^c^
		40	0.041 ± 0.002 ^b^	6.8 ± 0.6 ^b^	0.990	5.3 ± 0.4 ^b^	7.1 ± 0.4 ^b^
		50	0.051 ± 0.006 ^a^	10.4 ± 0.8 ^a^	0.993	9.7 ± 0.1 ^a^	10.2 ± 0.9 ^a^

Note: Mean ± standard deviation of nine replicates (n = 9). Different letters in the same column indicate significant differences between treatments, as determined by Tukey’s test (*p* ≤ 0.05), evaluated separately for each protease. US = ultrasound. k = hydrolysis reaction rate (min^−1^) at given temperature. DH∞ = Final hydrolysis degree (%). DH_45 min_ and DH_180 min_ = Hydrolysis degree at 45 and 180 min of hydrolysis, respectively (%).

**Table 2:** Protein solubility of pumpkin seed protein after 45 and 180 min of conventional and ultrasound-assisted hydrolysis at optimal temperature for each enzyme.

Enzyme	Hydrolysis	Hydrolysis Time (min)	Protein Solubility (%)				
			pH 2.0	pH 4.0	pH 6.0	pH 8.0	pH 10.0
Brauzyn^®^	Conventional hydrolysis	45	69 ± 3 ^cd^	23 ± 3 ^e^	17 ± 3 ^e^	56 ± 3 ^de^	84 ± 5 ^a^
		180	76 ± 3 ^b^	37 ± 3 ^cd^	32 ± 3 ^cd^	68 ± 4 ^bc^	88 ± 3 ^a^
	US assisted hydrolysis	45	77 ± 2 ^b^	37 ± 2 ^cd^	32 ± 4 ^cd^	70 ± 2 ^bc^	87 ± 3 ^a^
		180	81 ± 2 ^a^	46 ± 3 ^b^	41 ± 2 ^b^	77 ± 3 ^a^	88 ± 3 ^a^
Flavourzyme^®^	Conventional hydrolysis	45	63 ± 4 ^de^	18 ± 2 ^ef^	15 ± 3 ^e^	52 ± 3 ^e^	82 ± 4 ^a^
		180	68 ± 3 ^cd^	21 ± 2 ^e^	15 ± 4 ^e^	55 ± 3 ^e^	83 ± 4 ^a^
	US assisted hydrolysis	45	71 ± 5 ^bcd^	32 ± 2 ^d^	26 ± 3 ^d^	63 ± 4 ^cd^	86 ± 3 ^a^
		180	74 ± 4 ^bcd^	34 ± 2 ^d^	30 ± 4 ^cd^	67 ± 2 ^bc^	88 ± 3 ^a^
Neutrase^®^	Conventional hydrolysis	45	76 ± 3 ^b^	36 ± 3 ^d^	30 ± 4 ^cd^	66 ± 3 ^bc^	85 ± 4 ^a^
		180	79 ± 4 ^ab^	42 ± 2 ^bc^	36 ± 2 ^c^	72 ± 3 ^ab^	89 ± 3 ^a^
	US assisted hydrolysis	45	83 ± 2 ^a^	52 ± 2 ^a^	48 ± 3 ^a^	77 ± 3 ^a^	88 ± 4 ^a^
		180	84 ± 2 ^a^	54 ± 2 ^a^	50 ± 2 ^a^	79 ± 4 ^a^	90 ± 4 ^a^
Native PSP—non-hydrolyzed			60 ± 3 ^e^	14 ± 3 ^f^	12 ± 3 ^e^	50 ± 4 ^e^	81 ± 6 ^a^

Note: Mean ± standard deviation of nine replicates (n = 9). Different letters in column indicate significant difference (*p* ≤ 0.05) among treatments. US: ultrasound. PSP: pumpkin seed protein.

**Table 3:** In vitro antioxidant activity from the DPPH and ABTS assays of pumpkin seed protein after 45 and 180 min of conventional and ultrasound-assisted hydrolysis at the optimal temperature for each enzyme.

Enzyme	Hydrolysis	Hydrolysis Time (min)	DPPH Inhibition (%)	ABTS Inhibition (%)
Brauzyn^®^	Conventional hydrolysis	45	34 ± 2 ^c^	56 ± 2 ^c^
		180	37 ± 3 ^c^	58 ± 2 ^c^
	US assisted hydrolysis	45	43 ± 2 ^ab^	63 ± 3 ^bc^
		180	46 ± 2 ^a^	74 ± 4 ^a^
Flavourzyme^®^	Conventional hydrolysis	45	15 ± 2 ^f^	46 ± 2 ^de^
		180	21 ± 1 ^e^	47 ± 3 ^de^
	US assisted hydrolysis	45	28 ± 3 ^d^	58 ± 4 ^c^
		180	32 ± 3 ^cd^	67 ± 4 ^ab^
Neutrase^®^	Conventional hydrolysis	45	26 ± 3 ^d^	43 ± 1 ^e^
		180	28 ± 4 ^d^	42 ± 3 ^e^
	US assisted hydrolysis	45	38 ± 3 ^bc^	50 ± 3 ^de^
		180	40 ± 3 ^bc^	54 ± 2 ^c^
Native PSP—non-hydrolyzed			6 ± 1 ^g^	16 ± 1 ^f^

Note: Mean ± standard deviation of nine replicates (n = 9). Different letters in column indicate significant difference (*p* ≤ 0.05) among treatments. US: ultrasound. PSP: pumpkin seed protein.

**Table 4:** The mean particle size, polydispersity index (PDI) and zeta potential at pH 7.5 for protein hydrolysates obtained after 45 and 180 min of conventional and ultrasound-assisted hydrolysis at optimal temperature for each enzyme.

Enzyme	Hydrolysis	Hydrolysis Time (min)	Mean Particle Size (nm)	Polydispersity Index (PDI)	Zeta Potential (mV)
Brauzyn^®^	Conventional hydrolysis	45	566 ± 27 ^a^	0.911 ± 0.036 ^a^	−32.7 ± 1.3 ^a^
		180	302 ± 18 ^de^	0.643 ± 0.021 ^de^	−49.8 ± 1.9 ^cd^
	US assisted hydrolysis	45	419 ± 54 ^c^	0.792 ± 0.058 ^bc^	−47.3 ± 1.0 ^bc^
		180	223 ± 39 ^f^	0.512 ± 0.020 ^f^	−60.4 ± 1.7 ^gh^
Flavourzyme^®^	Conventional hydrolysis	45	377 ± 12 ^c^	0.736 ± 0.023 ^bcd^	−52.1 ± 2.0 ^de^
		180	168 ± 4 ^g^	0.592 ± 0.060 ^ef^	−58.8 ± 0.5 ^fgh^
	US assisted hydrolysis	45	211 ± 20 ^f^	0.618 ± 0.051 ^ef^	−57.6 ± 0.5 ^fg^
		180	124 ± 6 ^h^	0.574 ± 0.029 ^ef^	−62.4 ± 1.5 ^h^
Neutrase^®^	Conventional hydrolysis	45	513 ± 13 ^b^	0.838 ± 0.040 ^ab^	−44.8 ± 0.9 ^b^
		180	255 ± 30 ^ef^	0.607 ± 0.020 ^ef^	−55.4 ± 1.7 ^ef^
	US assisted hydrolysis	45	324 ± 24 ^d^	0.683 ± 0.018 ^cde^	−52.5 ± 1.1 ^de^
		180	224 ± 20 ^f^	0.600 ± 0.048 ^ef^	−61.7 ± 1.6 ^gh^

Note: Mean ± standard deviation of nine replicates (n = 9). Different letters in column indicate significant difference (*p* ≤ 0.05) among treatments. US: ultrasound.

**Table 5:** The UV intensity at 280 nm and the maximum fluorescence intensity, at pH 7.5, for protein hydrolysates obtained after 45 and 180 min of conventional and ultrasound-assisted hydrolysis.

Enzyme	Hydrolysis	Hydrolysis Time (min)	UV Intensity at 280 nm (au)	Maximum Fluorescence Intesinty (au)
Brauzyn^®^	Conventional hydrolysis	45	0.321 ± 0.043 ^fg^	5919 ± 73 ^de^
		180	0.354 ± 0.028 ^f^	7048 ± 171 ^b^
	US assisted hydrolysis	45	0.444 ± 0.034 ^e^	6698 ± 98 ^c^
		180	0.595 ± 0.032 ^bc^	8451 ± 201 ^a^
Flavourzyme^®^	Conventional hydrolysis	45	0.205 ± 0.034 ^h^	6156 ± 52 ^d^
		180	0.523 ± 0.042 ^cd^	6773 ± 89 ^bc^
	US assisted hydrolysis	45	0.564 ± 0.029 ^cd^	6725 ± 63 ^c^
		180	0.638 ± 0.020 ^b^	6612 ± 92 ^c^
Neutrase^®^	Conventional hydrolysis	45	0.285 ± 0.032 ^g^	4888 ± 95 ^f^
		180	0.310 ± 0.052 ^fg^	4674 ± 121 ^f^
	US assisted hydrolysis	45	0.511 ± 0.028 ^d^	5811 ± 94 ^e^
		180	0.742 ± 0.032 ^a^	8206 ± 172 ^a^

Note: Mean ± standard deviation of nine replicates (n = 9). Different letters in column indicate significant difference (*p* ≤ 0.05) among treatments. US: ultrasound.

**Table 6:** Percentage of the gain for each enzyme, under different sonication conditions, with the different attributes in the reactions performed at the optimum temperature.

Enzyme	T (°C)	US Treatment	Gains (%): DH∞	Hydrolysis Time (min)	Gains (%): Soluble Protein		Gains (%): Antioxidant Activity	
					pH 4	pH 6	DPPH	ABTS
Brauzyn^®^	60	Enzyme	8.6	45	61.3	83.8	26.6	4.3
				180	15.2	14.9	25.9	28.8
		Substrate	32.4	45	57.3	78.6	29.6	3.9
				180	20.6	24.7	32.2	15.5
		US-assisted	20.3	45	60.9	88.2	26.5	12.5
				180	24.3	28.1	24.3	27.6
Flavourzyme^®^	55	Enzyme	58.5	45	83.3	86.9	40.7	29.5
				180	66.7	94.8	38.2	65.7
		Substrate	48.7	45	44.4	72.4	71.3	29.5
				180	52.4	87.0	32.4	42.7
		US-assisted	63.9	45	77.8	73.3	86.7	26.1
				180	61.9	100.0	52.4	42.6
Neutrase^®^	50	Enzyme	42.2	45	39.8	53.8	39.8	28.6
				180	26.7	38.4	52.5	39.4
		Substrate	89.1	45	33.9	52.5	56.8	15.0
				180	25.1	36.4	39.4	19.0
		US-assisted	65.1	45	44.4	60.0	46.2	16.3
				180	28.6	38.9	42.9	28.6

## Data Availability

The data presented in this study are included in the article, any further inquiries can be directed to the corresponding author.
